# A young female physician with sudden buttock pain and inability to walk: A case report and literature review

**DOI:** 10.1097/MD.0000000000045137

**Published:** 2025-10-03

**Authors:** Xinyue Li, Qing Li, Kaixuan Zhang, Shunbao Li

**Affiliations:** a Third Department of Cardiology, Baoding No. 1 Central Hospital of Hebei Medical University, Baoding, Hebei, China; b Second Department of Cardiology, Baoding No. 1 Central Hospital of Hebei Medical University, Baoding, Hebei, China.

**Keywords:** infectious sacroiliitis, magnetic resonance imaging, methicillin-sensitive staphylococcus aureus, pyogenic sacroiliitis

## Abstract

**Rationale:**

Infectious sacroiliitis (ISI) is a rare infection of the sacroiliac joint, typically affecting adolescents and children. Its diverse and nonspecific clinical manifestations, along with often unremarkable early imaging findings, frequently lead to diagnostic delays and poor outcomes. This report presents a case of ISI caused by Staphylococcus aureus in a young female physician, aiming to enhance clinicians’ awareness of ISI in atypical populations, emphasize the importance of early imaging and microbiological testing in diagnosis, and provide insights into the clinical management of this rare condition.

**Patient concerns:**

A 29-year-old woman physician was admitted to our medical facility due to experiencing right hip pain for 5 days. The pain was characterized as constant and achy, not extending to other parts of the body, but intensifying notably when walking or bending. Resting provided some relief from the pain, and there was no significant discomfort on the left side. Additionally, she complained trouble walking and weakness in her right lower limb.

**Diagnoses:**

Accordingly, taking into account the patient’s medical background, physical assessment, and findings from admission tests, a diagnosis was made of methicillin-sensitive staphylococcus aureus bacteremia and right-sided ISI.

**Interventions:**

The patient received intravenous cefoperazone-sulbactam for a duration of 4 weeks as part of her anti-infection treatment regimen, with a dosage of 3.0 grams administered every 12 hours.

**Outcomes:**

Following 3 days of treatment, her pain levels decreased, enabling her to have some restricted movement. By the end of the first week, her body temperature normalized, her pain considerably lessened, and a subsequent blood culture came back negative. A follow-up magnetic resonance imaging of the sacroiliac joint 4 weeks later showed improvement in bone marrow edema specifically on the right side of the joint.

**Lessons:**

ISI is a rare condition that can be quite challenging to diagnose due to its vague and nonspecific symptoms. magnetic resonance imaging is considered the most reliable imaging technique for identifying ISI when there is suspicion of the condition. Early identification of the specific bacterial strain based on drug sensitivity testing is crucial for the effective clinical management of ISI and for predicting the patient’s prognosis.

## 1. Introduction

Infectious sacroiliitis (ISI) is a relatively rare disease that often presents with pain in the buttocks or sacral area.^[1]^ The clinical manifestations of ISI are similar to those of hip joint infection, intervertebral disc protrusion, or ankylosing spondylitis.^[2]^ Due to the varied symptoms of ISI, it is often misdiagnosed or diagnosed late in clinical practice. ISI can easily be confused with ankylosing spondylitis or lumbar disc herniation, making it important to understand the characteristics of this disease in order to reduce misdiagnosis and improve patient outcomes.^[3]^

The case presents a rare occurrence of ISI in a youthful female physician who was afflicted with staphylococcus aureus (SA). It emphasizes the challenges associated with diagnosing this disease in younger patients, underscores the potential for misdiagnosis, and points out the valuable role of magnetic resonance imaging (MRI) in detecting ISI in such cases. Based on our searching in literature, there are limited case reports regarding ISI resulting from SA. Additionally, we will examine previously documented instances of this condition.

## 2. Case report

A 29-year-old female physician was admitted to our hospital with complaints of right hip pain for 5 days. The patient was healthy in the past and busy with her work, and had a sedentary lifestyle. Her daily diet mainly consisted of vegetarian food. The patient experienced sudden onset right hip pain 5 days ago without any apparent cause. The pain was described as continuous and dull, not radiating to other areas, but becoming significantly worse with walking or bending. Resting helped alleviate the pain, with no significant pain on the other side, and no numbness, coolness, swelling, fever, or back pain in the lower limbs. She also complained difficulty walking and weakness in the right lower limb. Treatment including pain relief and anti-inflammatory measures did not provide significant relief for her, with persistent difficulty walking and weakness in the right lower limb, as well as difficulty turning over at night. On the day of admission, the patient developed a fever (temperature 38.4℃) with chills, but no nasal congestion, runny nose, cough, or sputum production. Upon admission, vital signs were as follows: temperature 37.9℃, pulse 96 beats per minute, respiration rate 18 breaths per minute, and blood pressure 98/75 mm Hg. She was thin in stature, with a BMI of 15.6kg/m^2^. She was wheeled into the ward in a wheelchair, displaying a pained expression but clear consciousness. Local tenderness was noted at the right sacroiliac joint, with significant painful limitation of movement in the right hip joint. She was unable to complete hip flexion and extension movements due to pain. The rest of the joints, muscle strength, and sensory functions in the right lower limb were normal. The pelvic compression test was positive right hip joint. Limited functional mobility in the right hip joint, forced position of hip flexion on the right side, hip flexion and internal/external rotation induced pain on the affected side, positive results on the 4-word test, bending restricted. Symptoms could also be elicited on the affected side with hip flexion on the left side.

**Figure 1.**
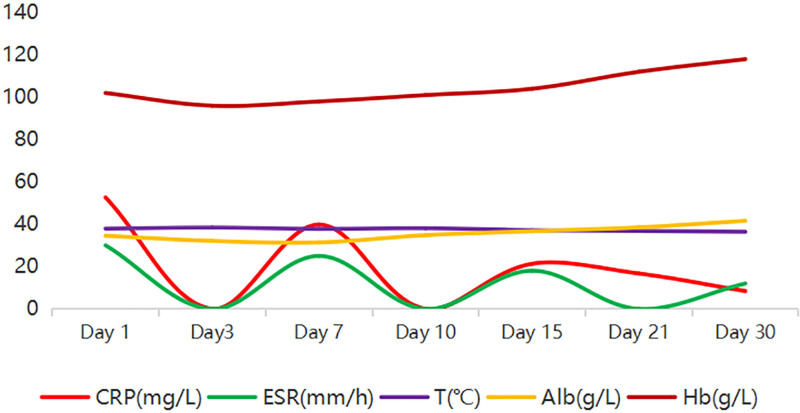
Trends of laboratory parameters and body temperature during treatment. Serial measurements of CRP (mg/L), ESR (mm/h), body temperature (T, °C), serum albumin (Alb, g/L), and Hb (g/L) are shown over the course of antibiotic therapy. Note the rapid decline in CRP and ESR, normalization of body temperature within 1 week, and gradual improvement in albumin and hemoglobin levels. CRP = C-reactive protein, ESR = erythrocyte sedimentation rate, Hb = hemoglobin.

**Figure 2.**
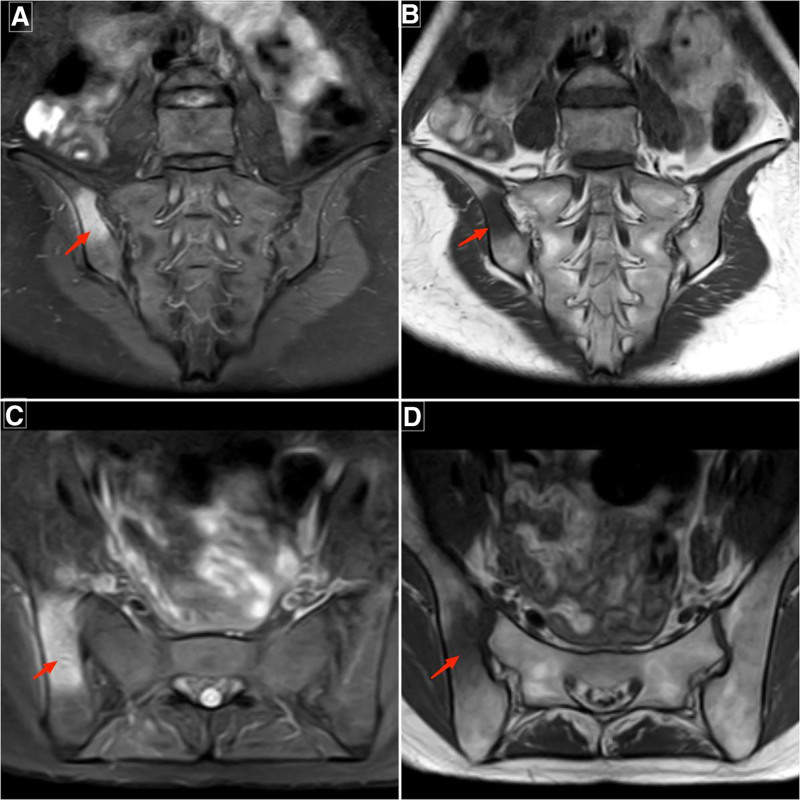
MRI of the sacroiliac joints on admission. (A and B) T2-weighted images with fat suppression show bone marrow edema, seen as patchy high signal intensity (arrows), beneath the articular surfaces of the right ilium and sacrum. Note the associated swelling of the adjacent gluteal and soft tissues, most prominent on the right.(C and D) Corresponding T1-weighted images demonstrate low signal intensity (arrows) in the same regions. The right sacroiliac joint space is narrowed. In contrast, the left sacroiliac joint appears normal in alignment, joint space width, and signal characteristics. MRI = magnetic resonance imaging.

Admission laboratory tests revealed a white blood cell (WBC) counts of 9.3 × 10^9^/L, hemoglobin level of 102 g/L, with neutrophils accounting for 0.897 of the total. The C-reactive protein (CRP) level was measured at 52.6 mg/L, while the procalcitonin level was 036ng/mL. Additionally, the erythrocyte sedimentation rate (ESR) was found to be 30 mm/h, indicating a possible infection. Her liver function test showed a mild decrease in albumin levels, with a value of 34.5g/L, while the rest were within normal ranges. The trend changes in the patient’s laboratory findings can be observed in Figure 1. Further testing for brucella antibody, tuberculosis screening, and immune system-related assays all yielded negative results. The human leukocyte antigen B27 (HLA-B27) test was also negative. Thyroid function, renal function, urinary test, tumor markers, and creatine kinase levels were all within normal ranges upon. Her head, chest, abdomen, and pelvic computed tomography (CT) scans showed no obvious abnormalities. Additionally, her cardiac ultrasound and lower extremity vascular ultrasound were also normal. A lumbar spine CT scan revealed a disc protrusion at the L4 to 5 and L5 to S1 level. On the third day of admission, a lumbar sacral joint of MRI revealed narrowing of the right sacroiliac joint space, bone marrow edema, prominent iliac bone surface, accompanied by swelling in the buttock muscles and soft tissues (Fig. 2A–D), suggestive of right sacroiliac joint inflammation. The blood culture results from the patient indicated the presence of methicillin-sensitive SA (MSSA), which was found to be sensitive to various antibiotics in vitro including penicillin, cefoxitin sulfabactam, and levofloxacin.

**Figure 3.**
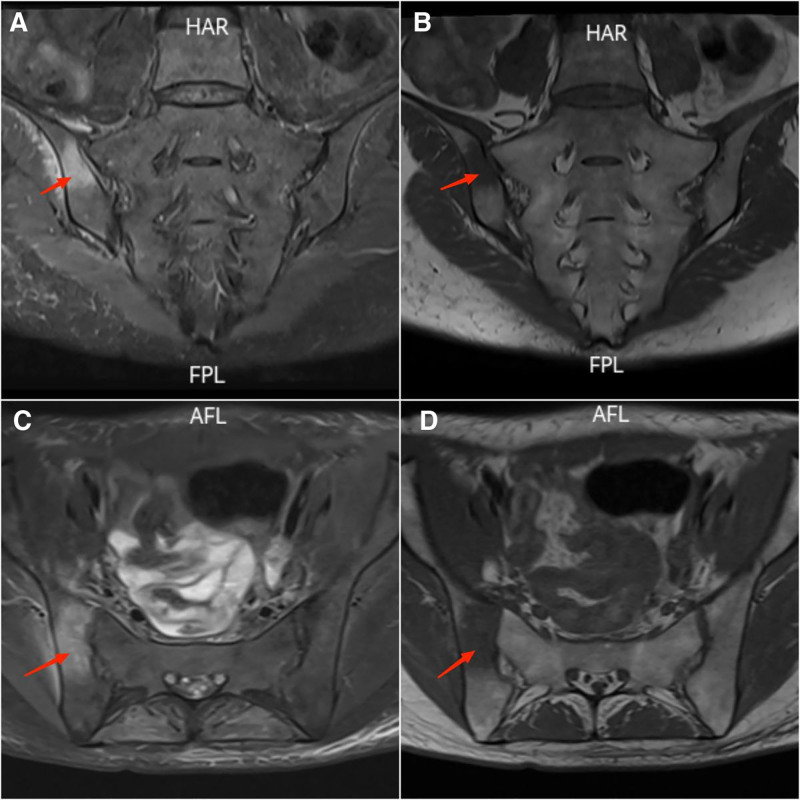
Follow-up MRI of the sacroiliac joints after 4 weeks of antibiotic therapy. (A and B) PD-weighted images demonstrate residual high signal intensity (arrow) on the iliac side of the right sacroiliac joint, indicating persistent bone marrow edema; however, the extent and intensity are significantly reduced compared to admission. The right joint space remains slightly narrowed. (C and D) Corresponding T1-weighted images show patchy low signal intensity (arrow) in the same region. Note the resolution of gluteal and soft-tissue swelling. The left sacroiliac joint remains normal in alignment, signal, and joint space. MRI = magnetic resonance imaging.

Thus, based on the patient’s medical history, physical examination, and admission test results, she was diagnosed with MSSA bacteremia and right-sided ISI. The patient showed significant improvement in both clinical manifestations and laboratory parameters after treatment with intravenous cefoperazone-sulbactam (3.0 g every 12 hours). After 3 days of treatment, her right hip pain markedly decreased, allowing limited mobility. By the first week, her body temperature had returned to normal (Fig. 1), pain was significantly relieved, and repeat blood culture was negative. Inflammatory markers declined rapidly: CRP decreased from 52.6 mg/L to within the normal range, ESR normalized from 30 mm/h, and WBC count with differentiation returned to normal. A follow-up MRI of the sacroiliac joint after 4 weeks of therapy demonstrated notable reduction in bone marrow edema on the right side compared to admission (Fig. 3A–D), with near-complete resolution of soft-tissue swelling. The patient completed 4 weeks of systemic antibiotic therapy and was discharged. During outpatient follow-up over one year, she reported no significant discomfort and exhibited good functional recovery.

## 3. Discussion

ISI is relatively rare in clinical practice, making it challenging to diagnose. The identification of ISI is frequently overlooked or delayed because of its ambiguous, nonspecific symptoms, coupled with its rarity as a diagnostic possibility.^[4]^ The most common types include pyogenic sacroiliitis (PSI), tuberculous sacroiliitis, and brucella-infected sacroiliitis.^[5]^ PSI has a prevalence of 1% to 2%, while tuberculous sacroiliitis and brucella-infected sacroiliitis are even rarer.^[6]^ ISI typically presents with acute onset, mainly affecting young individuals, with a peak incidence in the 20 to 30 age group, although cases have been reported in individuals of all ages.^[7]^ However, In the early stages, ISI is often misdiagnosed as sciatica, intervertebral disc inflammation, chronic pain syndrome, or spinal arthritis.^[8]^

The case involves a 29-year-old young female patient who experienced sudden onset of right hip pain, which progressively worsened, accompanied by difficulty walking and difficulty turning over at night. The patient was initially diagnosed with lumbar disc herniation. However, symptomatic treatment and physical therapy provided no significant improvement, and her condition continued to deteriorate. During the onset, there was a low fever, ultimately diagnosed through MRI indicating ISI on the right side. ISI in young adults without obvious triggers is clinically uncommon, with a very low incidence of sacroiliac joint infections.^[9]^ Diagnosis is challenging in clinical practice and can be easily confused with conditions like ankylosing spondylitis.

SA is the most common pathogen causing infectious arthritis, and this case also involved an infection by SA.^[10]^ The patient was admitted to our hospital with a fever and blood culture was taken, rapidly confirming the pathogen as SA. The most common risk factors for SA infection include intravenous drug use, pregnancy, trauma, acupuncture, endocarditis, sickle cell disease, immunosuppression, skin infections, respiratory or genitourinary tract infections.^[11–13]^ In children, one should be alert to a history of prior injuries, insect bites with severe skin reactions, atopic dermatitis, eczema, and acute furuncle disease. The majority of cases of female PSI are pregnancy-related, accounting for about 10%, and typically occur within 3 weeks postpartum or post-abortion.^[14]^ Early identification of risk factors based on these patient’s symptoms and signs can improve early diagnosis of ISI. A delayed diagnosis of SA infection may result in serious complications, including abscess formation, disseminated infection, sepsis, permanent disability, or even death. Definitive pathogen identification can be achieved through microbiological and molecular analysis of purulent specimens, including microscopy, culture, polymerase chain reaction, and next-generation sequencing.^[15]^ Live tissue biopsy of affected joints in patients with infectious sacroiliitis can significantly improve the pathogen detection rate. Despite this, diagnostic uncertainty may persist in a subset of cases.

The patient in this case was a healthy young female physician with a busy schedule, who primarily followed a vegetarian diet and had a habit of sedentary lifestyle. The patient had a slender physique, with a significantly low BMI. The patient presented with low BMI (15.6 kg/m²), vegetarian diet, and sedentary lifestyle, though their causal relationship to immunity requires cautious interpretation. Notably, she lacked classic risk factors (e.g., IV drug use, trauma, pregnancy). This case emphasizes that ISI may present acutely in young, healthy individuals without classic risk factors, a scenario prone to misdiagnosis.^[15]^ Admission laboratory findings revealed mild anemia and hypoalbuminemia, which may have compromised her immune function and potentially contributed to the onset of infectious sacroiliitis. The patient presented with a significantly low BMI, accompanied by mild anemia and hypoalbuminemia. Malnutrition may impair immune function through various mechanisms, including lymphocytopenia, dysregulated cytokine production, and compromised mucosal barrier integrity, potentially increasing susceptibility to pyogenic infections.^[5,6]^ Although a direct causal relationship between low BMI and ISI remains unproven, nutritional and immune status should be considered potential predisposing factors in young individuals lacking classic risk factors. PSI clinically often presents as more common in young women, with an acute onset, short duration, often accompanied by fever, typically unilateral, worsens with activity, and improves with rest, with clear localized tenderness.^[16]^ Therefore, the patient fits the clinical characteristics of PSI.

Laboratory findings are not specific for the diagnosis of ISI.^[17]^ Although elevations in neutrophils, ESR, and CRP are commonly observed in ISI, these findings are nonspecific and not diagnostic. In the present case, the patient demonstrated a normal WBC count, mildly elevated ESR, and a markedly elevated CRP level. CRP is an acute-phase protein produced directly in response to tissue injury and inflammation. ESR, on the other hand, measures the rate at which red blood cells settle in a blood sample. When acute-phase proteins like fibrinogen are present, red blood cells settle faster, leading to a higher ESR value. This serves as an indirect marker of acute-phase reactants and often takes longer to peak. In this instance, the patient was in the early stages of their illness, explaining the markedly elevated CRP level while the ESR was only slightly above normal limits.

Patients with ISI show evident bone destruction and widening of the joint space in CT imaging of the sacroiliac joint. The role of MRI in the diagnosis and differential diagnosis of ISI is becoming increasingly important. MRI allows detailed visualization of the complex anatomy of the sacroiliac joint and any associated soft-tissue abnormalities. Moreover, it accurately reveals pathological changes and provides critical information regarding inflammatory activity.^[18]^ MRI has a high sensitivity for detecting early inflammatory changes in the sacroiliac joint, with a diagnostic sensitivity for ISI as high as 100%.^[19]^ In this case, the patient presented with typical signs of ISI on MRI imaging, having developed symptoms within the past week. MRI is essential for assisting in the diagnosis of ISI, with approximately half of the cases diagnosed through MRI showing adjacent formation of an abscess. CT is advantageous for highlighting bone destruction and can guide sacroiliac joint aspirations. Our successful pathogen identification via blood culture represents a fortuitous occurrence. The literature consistently reports low diagnostic yield of blood cultures in ISI, with positivity rates frequently below 50% (as low as 25% in some series).^[6,15]^ In contrast, image-guided joint aspiration demonstrates superior diagnostic efficacy, achieving yields of 80% to 90%.^[16]^ Early microbiological diagnosis remains vital for treatment. However, due to the limited sensitivity of blood cultures, image-guided joint aspiration is recommended as the first-line diagnostic modality when blood culture results are negative.

Prompt and immediate antibiotic treatment was initiated in this patient upon her diagnosis of ISI. Antibiotics serve as the cornerstone of treatment and are typically targeted specifically against Gram-positive pathogens, which are the predominant culprits in PSI, accounting for approximately 80% of cases.^[20]^ In this case, the patient was diagnosed with right-sided PSI caused by MSSA. Targeted treatment with antibiotics for the specific pathogen led to significant improvement in local symptoms after 1 week. Follow-up tests showed a return to normal levels of inflammatory markers CRP and ESR, and repeat MRI of the sacroiliac joint at 4 weeks showed improvement. Symptoms continued to improve after discontinuation of medication, and the patient did not undergo needle aspiration, surgical drainage, or biopsy. Six months of follow-up showed no discomfort. It is noteworthy that abnormal MRI findings in PSI can persist for months after clinical improvement. Therefore, complete radiographic resolution should not be solely relied upon as a criterion for discontinuing treatment. There is a lack of consensus in the literature regarding the optimal duration of treatment for PSI, with a wide range of recommendations provided.^[21,22]^ However, most sources suggest that a minimum of 4 weeks of antibiotic therapy is necessary.^[23]^ When specific culture results are unavailable to guide therapy, empiric antibiotic coverage should target Gram-positive bacteria, as they account for most infections associated with invasive spinal instrumentation. Patients presenting with signs of sepsis should receive broad spectrum antibiotic coverage. It is crucial to promptly initiate appropriate antibiotic therapy, as untreated ISI can lead to complications such as abscess formation, osteomyelitis, and sepsis.

While our case demonstrated excellent short-term outcomes, the long-term prognosis of ISI requires realistic counseling. A 5-year cohort study of 62 PSI patients revealed 43.5% developed persistent mechanical lumbosacral pain.^[6]^ This pain primarily stems from structural joint damage and correlates significantly with initial disease severity and treatment delay.^[23]^ We advise biannual visual analogue scale pain assessment and sacroiliac stress testing for ≥ 3 years post-recovery. High-risk patients (e.g., BMI < 18.5 or initial CRP > 50mg/L as in this case) should receive prophylactic physical therapy.

Our investigation covered the cases of PSI caused by SA reported in the past decade (2014-present). Following a systematic literature search on PubMed, Embase, and Web of Science online databases with “pyogenic sacroiliitis” and “SA” keywords, we have identified 25 cases of PSI. Among these ones, 9 cases were attributed to SA in female patients (Table 1).^[3–5,19,20,24–26]^ This suggests that PSI caused by SA is quite rare. Notably, nearly all reported cases were female patients. Besides, the patients’ ages ranged from 13 months to 72 years, with an average age of 27 years. Among them, 5 cases (55.5%) involved the right sacroiliac joint, 3 case (33.3%) involved the left, and 1 case (11.2%) involved both joints. In terms of clinical symptoms, all patients presented with varying degrees of fever, with temperatures ranging from 37.6 to 39.2°C. Most patients experienced hip and buttock pain, sometimes accompanied by lower back pain. Four patients had no prior history of illness, while one had a history of systemic lupus erythematosus and another was in the late stage of pregnancy. All reported cases were diagnosed in the early stages based on imaging modalities such as MRI, Bone scan, X-ray, and CT. The causative agent of PSI was confirmed as SA through culture of puncture fluid and blood. Laboratory tests revealed significantly elevated inflammatory markers in these patients. Treatment strategies included abscess drainage, antibiotic therapy (e.g., cefotaxime, clindamycin, or fosfomycin), surgical debridement, and primary arthrodesis. Owing to early diagnosis and intervention, only 1 mortality occurred; the remaining patients achieved a favorable prognosis. Hence, S. aureus sacroiliitis carries an excellent prognosis (nearly 90% survival). Surgery is critical for abscess/necrosis, while extreme CRP (>2000 mg/dL) does not preclude recovery with timely intervention.

**Table 1:** Previous reports of pyogenic sacroiliitis caused by *Staphylococcus aureus* in female patients.

Author	Year	Age	Affected joint	Temperature (^o^C)	Clinical feature	Past medical history	Imaging test	ESR (mm/h)	CRP (mg/dL)	Treatment	Prognosis
Leroux et al^[24]^	2015	13mo	R	37.6	Weight loss in the right lower extremity	Healthy	MRI + Bone scan + X-ray + CT	NR	380	IV cefotaxime	Improved
Imagama et al^[20]^	2015	37yr	R	38.4	Right hip pain	Healthy	MRI + Bone scan + X-ray + CT	NR	2200	Drainage of abscess cavity + IV antibiotic and loanword	Improved
Kreps et al^[3]^	2014	22yr	R + L	39	Severe right gluteal and pelvic pain.	Healthy	X-ray + MRI + CT	99	19.74	IV clindamycin	Improved
Millwala et al^[4]^	2015	33yr	R	37.7	Right sciatica	Late pregnancy	MRI + CT	105	192.5	IV nafcillin	Improved
Chebbi et al^[5]^	2014	52yr	R	38.6	Acute pain in right hip and buttock	SLE	X-ray + MRI + CT	108	864	IV cefotaxime with fosfomycin	Improved
Passaplan et al^[19]^	2020	16yr	L	NR	Low back and left hip pain	Healthy	MRI + CT	NR	3180	Surgical debridement and primary arthrodesis	Improved
Bayala et al^[25]^	2025	55yr	R	NR	Gluteal pain and weight-bearing difficulty	Pulmonary embolism	MRI	NR	29.5	Amoxicillin and Ofloxacin	Improved
Bayala et al^[25]^	2025	72yr	L	39.2	Gluteal pain and weight-bearing difficulty	Chronic low back pain	MRI	NR	50.4	Amoxicillin/clavulanate and Gentamicin	Died
Pathrot et al.^[26]^	2025	25yr	L	NR	Positive straight leg raise and inability to walk	Postpartum day 22	MRI	120	23.79	Surgical drainage and Vancomycin	Improved

CPR = c- reactive protein, CT = computed tomography, ESR = erythrocyte sedimentation rate, F = female, IV = intravenous, L = left, m = month, MRI = magnetic resonance imaging, NR = not reported, R = right, SLE = systemic lupus erythematosus, yr = year.

In contrast to most previously reported cases of SA sacroiliitis, which occur in patients with recognized risk factors such as pregnancy, trauma, or immunosuppression, our case involved a otherwise healthy young female with no classic risk factors. This emphasizes that ISI should remain a diagnostic consideration even in individuals without traditional risk factors. Furthermore, the unexpected utility of blood culture in this case – contrasting with its typically low yield (often below 50%) as reported in the literature – underscores the importance of pursuing image-guided joint aspiration when ISI is strongly suspected, even if blood cultures are negative. Thus, our case reinforces the necessity of early, multimodal diagnostic approaches in atypical populations.

This case offers several important lessons for clinical practice: First, it underscores that ISI should not be ruled out even in young, otherwise healthy individuals without classic risk factors (e.g., IV drug use, trauma, or pregnancy). Second, in patients presenting with acute unilateral buttock pain and functional impairment, ISI should be considered early in the differential diagnosis, and MRI should be pursued proactively – even if initial imaging is unremarkable – to facilitate early detection. Third, the case highlights the importance of obtaining microbiological confirmation (e.g., via blood culture or image-guided joint aspiration) to guide targeted antibiotic therapy and improve outcomes, even while initiating empirical treatment. In summary, this case reinforces the need for clinicians to maintain a high index of suspicion for infectious etiologies when evaluating young adults with atypical musculoskeletal pain, even in the absence of traditional risk factors. A multimodal diagnostic approach combining advanced imaging and laboratory studies is essential for early diagnosis and management.

This report has several limitations. First, the follow-up period was relatively short (outpatient follow-up for 1 year), which precluded assessment of long-term functional recovery or potential sequelae, such as chronic pain or limited joint mobility. Second, the lack of systematic long-term imaging follow-up (e.g., MRI) prevented objective confirmation of complete radiological resolution of sacroiliac inflammation. Furthermore, as a single-center case report, the generalizability of the findings is limited. Future studies should incorporate longer-term imaging and functional assessments to better understand the natural history and treatment outcomes of the disease.

## 4. Conclusions

ISI is a rare disease that can be challenging to identify due to its nonspecific signs and symptoms. Moreover, laboratory abnormalities may not always be evident or may be minor. Standard imaging techniques like radiographs and CT scans often fail to show any abnormalities in the early stages of ISI. Healthcare providers should consider ISI as a potential diagnosis in patients experiencing lumbosacral pain, particularly when the signs, symptoms, and lab results are inconclusive. MRI is considered the most effective imaging tool for detecting ISI when there is suspicion of the condition. A definitive diagnosis of ISI is typically confirmed through a combination of biopsy and joint fluid culture. Additionally, SA is a common pathogenic bacterium in PSI. Early selection sensitive bacterial based on drug sensitivity results is crucial for clinical treatment of PSI and prognosis.

## Acknowledgments

We acknowledge the contributions of the colleagues in Baoding No. 1 Central Hospital that aided the efforts of the authors.

## Author contributions

**Conceptualization:** Shunbao Li.

**Data curation:** Qing Li.

**Formal analysis:** Qing Li.

**Funding acquisition:** Shunbao Li.

**Project administration:** Kaixuan Zhang, Shunbao Li.

**Resources:** Kaixuan Zhang, Shunbao Li.

**Software:** Kaixuan Zhang.

**Writing – original draft:** Xinyue Li.

**Writing – review & editing:** Xinyue Li.

## References

[1] Wang Y Geng S Lin Z. Clinical and imaging characteristics of 135 cases of infectious sacroiliitis: a retrospective cohort study in China. Clin Rheumatol. 2025;44:1337–44. 39862334 10.1007/s10067-024-07278-8

[2] Matt M Denes E Weinbreck P. Infectious sacroiliitis: retrospective analysis of 18 case patients. Med Mal Infect. 2018;48:383–8. 29692328 10.1016/j.medmal.2018.02.001

[3] Weber U Jurik AG Zejden A. Frequency and anatomic distribution of magnetic resonance imaging features in the sacroiliac joints of young athletes: exploring “Background Noise” toward a data-driven definition of sacroiliitis in early spondyloarthritis. Arthritis Rheumatol. 2018;70:736–45. 29430880 10.1002/art.40429

[4] Kapuczinski A Place S Filleul O Villers C. Successful treatment of spondylodiscitis and infectious sacroiliitis due to listeria monocytogenes using meropenem as salvage therapy. Eur J Case Rep Intern Med. 2022;9:003177. 35402343 10.12890/2022_003177 PMC8988489

[5] Putilina MV Ivanova MP Petrikeeva AE Berns SA. Difficulties in diagnosing sacroiliitis in young patients. Zh Nevrol Psikhiatr Im S S Korsakova. 2020;120:117–26. Russian. 32929934 10.17116/jnevro2020120081117

[6] Kucera T Brtkova J Sponer P. Pyogenic sacroiliitis: diagnosis, management and clinical outcome. Skeletal Radiol. 2015;44:63–71. 25231169 10.1007/s00256-014-1999-y

[7] Brtalik D Pariyadath M. A case report of infectious sacroiliitis in an adult presenting to the emergency department with inability to walk. J Emerg Med. 2017;52:e65–8. 27866812 10.1016/j.jemermed.2016.10.022

[8] Diacinti D Gioia C Vullo F Cannavale G Catalano C Valesini G. Magnetic resonance imaging findings of infectious sacroiliitis associated with iliopsoas abscess: a case report in a young male. Reumatismo. 2018;70:264–7. 30570246 10.4081/reumatismo.2018.1071

[9] Nagashima M Watanabe N Okui Y. Infectious sacroiliitis due to group A streptococcus infection during pregnancy: a case report. J Med Case Rep. 2022;16:55. 35144688 10.1186/s13256-022-03271-4 PMC8832776

[10] Monteiro R Cabrera JA Salvador R Pereira C Monteiro M. Infectious sacroiliitis as a rare postpartum complication: a case report. Cureus. 2024;16:e54621. 38524026 10.7759/cureus.54621 PMC10959255

[11] Darvishi M Rashidi S Abazari S. Tuberculous sacroiliitis after a penicillin injection: a case report. Eur J Case Rep Intern Med. 2020;7:001495. 32523913 10.12890/2020_001495 PMC7279915

[12] Araos-Baeriswyl E Araya A Luco V Monsalve X. Infectious sacroiliitis caused by Bartonella henselae in an immunocompetent adult: an unusual case. Enferm Infecc Microbiol Clin (Engl Ed). 2021;39:257–8. 33077343 10.1016/j.eimc.2020.07.005

[13] Çolak AF Yazar B Bucağa TM. A rare case presentation of septic sacroiliitis caused by staphylococcus xylosus and complicated with abscess formation: a case report. Diagn Microbiol Infect Dis. 2024;109:116290. 38643676 10.1016/j.diagmicrobio.2024.116290

[14] Lee A Gupta M Boyinepally K Stokey PJ Ebraheim NA. Sacroiliitis: a review on anatomy, diagnosis, and treatment. Adv Orthop. 2022;2022:3283296. 36620475 10.1155/2022/3283296 PMC9812593

[15] Arcângelo J Ramos SN Alves P. Pyogenic sacroiliitis: lessons learned from an atypical case series. An Pediatr (Engl Ed). 2019;91:42–6. 31130517 10.1016/j.anpedi.2018.07.017

[16] Kheder EM Sharahili HH Albahrani SY. Perinatal sacroiliitis diagnostic challenges. SAGE Open Med Case Rep. 2021;9:2050313X211052442. 10.1177/2050313X211052442 PMC8516381 34659772

[17] Tokuyama Y Yamada H Shinozuka K Yunoki T Ohtsuru S. Pyogenic sacroiliitis caused by Salmonella schwarzengrund in a young healthy woman: a case report and literature review. Int J Emerg Med. 2023;16:21. 36941606 10.1186/s12245-023-00496-y PMC10026423

[18] Østergaard M. MRI of the sacroiliac joints: what is and what is not sacroiliitis? Curr Opin Rheumatol. 2020;32:357–64. 32453038 10.1097/BOR.0000000000000718

[19] Passaplan C Simonin A Maestretti G Gautier E. Management of instability following pyogenic sacroiliitis: technical case report. Case Rep Orthop. 2020;2020:3409306. 32181037 10.1155/2020/3409306 PMC7066396

[20] Imagama T Tokushige A Sakka A Seki K Taguchi T. Postpartum pyogenic sacroiliitis with methicillin-resistant Staphylococcus aureus in a healthy adult: a case report and review of the literature. Taiwan J Obstet Gynecol. 2015;54:303–5. 26166346 10.1016/j.tjog.2013.10.044

[21] Yamada Y Yamaguchi H Ito Y Takeuchi N Kasai M. Pyogenic sacroiliitis caused by pneumococcal serotype 16F in a child. Pediatr Int. 2019;61:1267–8. 31814206 10.1111/ped.14034

[22] Thomas S Haque S Radia T. A case report of pyogenic sacroiliitis in a 9-month-old child. Arch Clin Cases. 2022;9:108–11. 36176492 10.22551/2022.36.0903.10213 PMC9512130

[23] Slobodin G Rimar D Boulman N. Acute sacroiliitis. Clin Rheumatol. 2016;35:851–6. 26847855 10.1007/s10067-016-3200-6

[24] Leroux J Bernardini I Grynberg L. Pyogenic sacroiliitis in a 13-month-old child: a case report and literature review. Medicine (Baltim). 2015;94:e1581. 10.1097/MD.0000000000001581 PMC4620820 26496260

[25] Bayala YLT Tinni IA Kaboré F. Infectious pyogenic sacroiliitis following acupuncture: a series of three cases and review of the literature. Acupunct Med. 2025;43:114–9. 40119763 10.1177/09645284251327200

[26] Pathrot D Nachappa AA Mannual S M SK. Postpartum pyogenic sacroiliitis masquerading as sciatic neuropathy. BMJ Case Rep. 2025;18:e263417. 10.1136/bcr-2024-263417 39880480

